# Effect of Maternal Nutrition on Cognitive Function of Children

**DOI:** 10.3390/nu13051644

**Published:** 2021-05-13

**Authors:** Victoria Arija, Josefa Canals

**Affiliations:** 1 Research Group in Nutrition and Mental Health (NUTRISAM), Universitat Rovira i Virgili, 43204 Reus, Spain; josefa.canals@urv.cat; 2 Research Center for Behavior Assessment (CRAMC), Universitat Rovira i Virgili, 43002 Tarragona, Spain; 3 Institut d’Investigació Sanitària Pere Virgili (IISPV), 43007 Reus, Spain

The intrauterine environment and, specifically, the nutritional status of the mother are crucial factors that can have short and long-term consequences on the health and disease risk of an unborn child. The fetal brain is highly vulnerable to nutritional alterations in pregnancy because of the role they play in programming its development and consequent mental processes. Although the beneficial effects of adequate nutritional status in pregnancy have been studied extensively over the last decade, many questions remain open, such as which are the most vulnerable periods of pregnancy, what are the effects of specific nutrients, and what levels of nutrients are required to improve cognitive development in the population. In this special issue, we focus on the effect of maternal nutrition on the cognitive function of children; specifically, we look at two systematic reviews and four empirical studies that analyzed the effects of type of diet and certain nutrients such as choline, iron, vitamin D, long-chain polyunsaturated fatty acids and folate.

Derbyshire & Obeid [1] present a systematic review of the role of choline in neurological development and brain function. Although it is a not well studied nutrient, it is known that inadequate choline can disrupt fetal brain development and lead to lifelong deficits. Findings suggest that supplementing the mother’s or child’s diet with choline over the first 1000 days of life may support normal brain development and protect against neural and metabolic insults. Likewise, the evidence from animal studies showed an improvement in neural and cognitive functioning.

The systematic review by McCann et al. [2] focuses on iron, which is essential for many processes in brain development and whose deficiency worldwide is particularly prevalent among pregnant women, infants and young children. They looked at several studies which examined the relation between iron deficiency or iron supplementation, at several timings of exposure, and neurodevelopment. They did not find a clear relationship between iron status during pregnancy, at 0–6 months, at 6–24 months, or at 2–4 years of age and motor, cognitive or socio-emotional development. They concluded that despite good mechanistic evidence for the role of iron in brain development, evidence regarding the impact of iron deficiency or iron supplementation on early development in humans is inconsistent.

In a rat model, Rivera et al. [3] investigated whether a high-caloric palatable diet given to the mother and/or to the offspring during the perinatal and/or postnatal period might dysregulate emotional behavior and prefrontal cortex function in the offspring at adult age. To this end, they studied the long-term anxiety responses and expression of glutamatergic and GABAergic receptors and the endocannabinoid system. Male animals born from mothers fed the palatable diet and who continued with this diet after weaning exhibited anxiety associated with an overexpression of glutamate receptors in the prefrontal cortex and a reduced expression of the endocannabinoid system. The authors concluded that a hypercaloric maternal diet induces sex-dependent anxiety, which is associated with alterations in both glutamatergic and cannabinoid signaling in the prefrontal cortex, which in turn are accentuated with the continuation of the palatable diet during the life of the offspring.

Campoy et al. [4] studied how providing mothers with supplements of certain foods/nutrients affected brain development and mental performance in offspring (NUHEAL study). The main result of their study was that supplementation with fish oil (FO), 5-methyltetrahydrofolate (5-MTHF), FO + 5-MTHF, or placebo did not affect processing speed in children aged 7.5 to 9 years of age. However, in secondary exploratory analyses, they observed that 9-year-old children born to mothers who had a higher AA/DHA ratio and who were heterozygous for FADS1 rs174556 showed significantly better performance in processing speed. They also observed negative associations between processing speed and maternal tHcy levels. Therefore, the authors concluded that quantitatively adequate and individualized prenatal supplementation for mothers can promote brain development and mental performance in their offspring. Controversial results have been reported in several studies regarding long-term effects of n-3 or n-6 LC-PUFAs supplementation during pregnancy on the child’s later neurodevelopment, and some studies have found that higher DHA/AA ratio was beneficial for infant development. However, data in the current study indicates the importance not only of DHA (n-6 LC-PUFA) but also its equilibrium with AA (n-3 LC-PUFA), supporting that early availability of AA during prenatal period is positively associated to cognitive performance in later childhood through an increase of white matter volume and better integrity. Moreover, the authors also observed negative associations between processing speed and maternal tHcy levels. Therefore, they concluded that quantitatively adequate and individualized prenatal supplementation for mothers can promote brain development and mental performance in their offspring.

To determine the benefit for maternal-child health of maintaining an optimal fatty acid (FA) profile during pregnancy, Aparicio et al. [5] studied the environmental factors that were related to levels of saturated FA, monounsaturated FA and omega 3 and omega 6 polyunsaturated FA at the beginning and end of pregnancy in a sample of healthy pregnant women from the Spanish Mediterranean area (ECLIPSES study). Adjusted multivariate analyses for several maternal factors (educational level, age, BMI, smoking, alcohol consumption, physical activity, food consumption) showed that higher educational levels, older age, greater consumption of fish and shellfish, and/or not smoking during pregnancy were associated with higher concentrations of eicosapentaenoic acid (EPA) and docosahexaenoic acid (DHA), and consequently the indices for n-6/n-3 and arachidonic acid (AA)/EPA were reduced. These associations were mostly observed at the beginning and at the end of gestation. However, none of these lifestyle factors were related to levels of saturated FA or monounsaturated FA.

In the same sample of pregnant women from the ECLIPSES study, the same research group [6] analyzed the effect of vitamin D status at the beginning and end of pregnancy on infant neurodevelopment (cognitive, linguistic and motor skills), which they evaluated at 40 days after delivery using Bayley Infant Development Scales III. Maternal vitamin D deficiency (<30 nmol/L) in the first trimester of pregnancy was found to predict worse performance in cognitive and language skills, independent of several confounding factors. In addition, mothers with severely deficient levels of vitamin D (<20 nmol/L) had babies with worse language performance and lower motor skills. Therefore, given the damage that maternal vitamin D deficiency causes to the neurological development of the baby, the authors recommend that the potential benefits of vitamin D supplementation during pregnancy be investigated and evaluated.

## Data Availability

Not applicable.
